# How to reach people who do not want to be reached: psychosocial counseling for school-dropouts in vocational training

**DOI:** 10.3389/fpsyg.2023.1112919

**Published:** 2023-12-18

**Authors:** Julian Valentin Möhring, Méline Wölfel, Burkhard Brosig

**Affiliations:** ^1^Department of Sociology, Faculty of Social and Cultural Sciences, Distance University, Hagen, Germany; ^2^Department for Child and Family Psychosomatics, Faculty of Medicine, Clinic for General Pediatrics and Neonatology of the University Clinic, Giessen, Germany; ^3^Horst Eberhard Richter Institute for Psychoanalysis and Psychotherapy, Giessen, Germany

**Keywords:** psychosocial counseling, emerging adults, vocational training, identity, defense mechanisms

## Abstract

Emerging adulthood without vocational training concerns young people from difficult social backgrounds who are often not adequately reached by therapeutic help. Difficult and traumatic experiences with therapeutic institutions are common to many of these young people in addition to a long lasting, unsatisfying patient-career. Without professional support from the therapeutic field, vocational qualification measures often cannot meet the needs of young people with inner conflicts. A counseling center for people with mental health problems was set up in 2010 as a link between professional support and a therapeutic setting. This article critically examines the importance of counseling for improving the personal situation of participants in vocational qualification measures on a descriptive level. We take a perspective on identity development and defense mechanisms in the thought of Vaillant and Erikson. Both theories focus on the social embeddedness of psychological processes. This theoretical background helps to understand young peoples’ situations and difficulties. The unique setting of the counseling center that aims to match the particular needs of these young people is presented. Thus a low-threshold, destigmatising and flexible setting should provide better access to psychosocial support for participants in vocational qualification measures. Opportunities and limits of the concept are discussed.

## 1 Introduction

We are not getting anywhere!

This statement stood at the beginning of the project to be described here, thus gave the starting signal for considerations on how young people in their emerging adulthood (Arnett, 2004) could be supported in their personal development. The board of a vocational training institute (“Jugendwerkstatt Giessen”) reported increasingly frequent difficulties in the vocational support of these young people. The teaching of technical skills is not successful as the psychological stress of the training participants affects them. The participants are considered difficult to reach, even though they seem to be in contact with the vocational training center. Many enroll in vocational training just to avoid financial sanctions from the social welfare system. This is the case for those who are dependent on social benefits but remain at home, even though they are capable of working. In summary, even those young people who are reached by a vocational training center remain difficult to reach for educational efforts, such as catching up on schooling. High levels of absenteeism, a lack of health-related self-care, and substance abuse are typical. Behind all of this, a problematic relationship with the family of origin becomes apparent, or a disrupted socialization at home is reported. Psychiatric and psychotherapeutic pre-treatment is often reported as being a traumatic experience: never again. Therefore, the project aims to develop a psychotherapeutic approach that also addresses those who consciously refuse to go through with it, but latently demand it by behaving as they do. In some cases, they can thus make a new attempt and try out a therapeutic setting.

### 1.1 Concept development

In the 1970s, a student movement was formed at Giessen University that combined psychoanalytic approaches with neighborhood work in a socially disadvantaged area of the city (cf. Brosig et al., 2017). Various group and individual discussions were offered and supervised by Horst Eberhard Richter, who held the Giessen Chair of Psychosomatics. The concerns of the residents of this neighborhood were discussed in a community center under psychoanalytic supervision. The residents were involved in political and social processes to improve their situation. Thus, important progress has been made on both self-efficacy and neighborhood amenities. In this tradition, the project presented in this article is based on a request from a vocational training institute to a psychosomatic department. The objective of this project is an intervention for young people in transition from school to work. Counseling began in 2010 with one psychosocial counselor and continues today with three counselors.

### 1.2 Theoretical background

Erikson (1994), in his groundbreaking work on “Identity and the Life Cycle” (1994), emphasizes the close connection between psychological development, its related conflicts, and the individual’s effectiveness in the external social world. He describes stages of identity development and presents results in the acquisition of psychosocial competencies: school graduation, sexual-partner commitment, assuming family roles, and the development of professional competencies are thus inextricably linked and shaped by psychological processing of one’s inner conflict issues. Erikson formulates a concept of the life cycle, which allows to relate present psychological difficulties to a specific phase of life, in which the problems that a client brings to a counseling session usually occur. This concept has been widely accepted, especially regarding the identity-forming phase of adolescence (Marcia, 1966), late adolescence (Blos, 1962), and the differentiation of adolescence and emerging adulthood (Arnett, 2004, Silva, 2013), and thus represents an important framework for psychosocial counseling as a focal therapeutic concept. Arnett (2004) identifies emerging adulthood as a phase that begins after adolescence and the completion of puberty, independence (in many cases moving out of the parental home), and compulsory schooling, but before young adulthood. The clients of the psychosocial counseling are between 18 and 27 years of age, so they are in the stage of the emerging adulthood. However, in view of the clients’ often chronic inner conflicts, which are rooted in the earlier stages of life, the motive of inner consistency emphasized by Erikson with regard to personal development is even more important.

On the other hand, the work of Vaillant (2012) on a healthy, mature defense shows that mental health, the ability to activate resources and creativity, strengthens the overall work performance in one’s own life. Defense mechanisms refer to innate involuntary regulatory processes that enable individuals to reduce cognitive dissonance and minimize sudden changes in the internal and external environment by altering perception. Defense mechanisms can distort perception of: subject, object, idea, and emotion (Vaillant, 1971). They can also affect the relationship between self and object (Vaillant, 1971). Sigmund and Anna Freud (Freud, 1937) outlined important characteristics of defense mechanisms: they are an important means of conflict and affect management and can be both adaptive and pathological. Immature defenses primarily underlie personality disorders. Skodol and Perry (1993) described the following immature defenses: devaluation, idealization, and omnipotence on a minor perception distortion level; denial, rationalization, projection on a denial level; splitting, projective identification, autistic fantasy on a major distortion level; acting out, hypochondriasis, provocative passive aggression on an action level. In a review of 50 years of research on mental defenses, Vaillant (2012) showed that failure scenarios are associated with immature defenses: development of chronic mental disorders, high burden of physical illness, increased accidents, and even premature death, often by suicide (Vaillant, 1992). This is often associated with occupational failure. The association of low adult social class with immature defensive use appeared to be a result, not a cause, of the immaturity of the defensive style (Vaillant, 1992). The relationship between defense and adult socioeconomic status was empirically demonstrated by Snarey and Vaillant (1985). The study by Plaude and Raščevska (2011) supports the idea that unemployed individuals may have less mature defense mechanisms than employed individuals. Focal therapy and the biographical approach to psychosocial counseling are the subject of the next section.

## 2 Context

### 2.1 Setting

The psychosocial counseling service is aimed at young people in their adolescence and emerging adulthood with problems in the transition from school to work, who are undergoing a one-year qualification measure at a training institute. The counseling concept was developed in collaboration between a clinical unit for family psychosomatics and a professor of rehabilitation pedagogy. The project adopts a conceptual approach that takes an integrated view of the three areas of vocational, psychological and health promotion. When the fields of vocational pedagogy, developmental psychology and medicine are intertwined, individual support services can be initiated and provided on an interdisciplinary basis. The cornerstones of the service are voluntary participation and confidentiality about the content of the counseling sessions, immediate accessibility through multi-professional cooperation and local placement of the service (door to door), and avoidance of stigmatization through the use of a psychosocial oriented service. Participants in this program face barriers to integration into the labor market. For this reason, low thresholds are important for the counseling service. Therefor the counseling room is located inside the vocational training institute and the counselors are available immediately when a participant is willing to take advantage of the counseling service.

**FIGURE 1:**
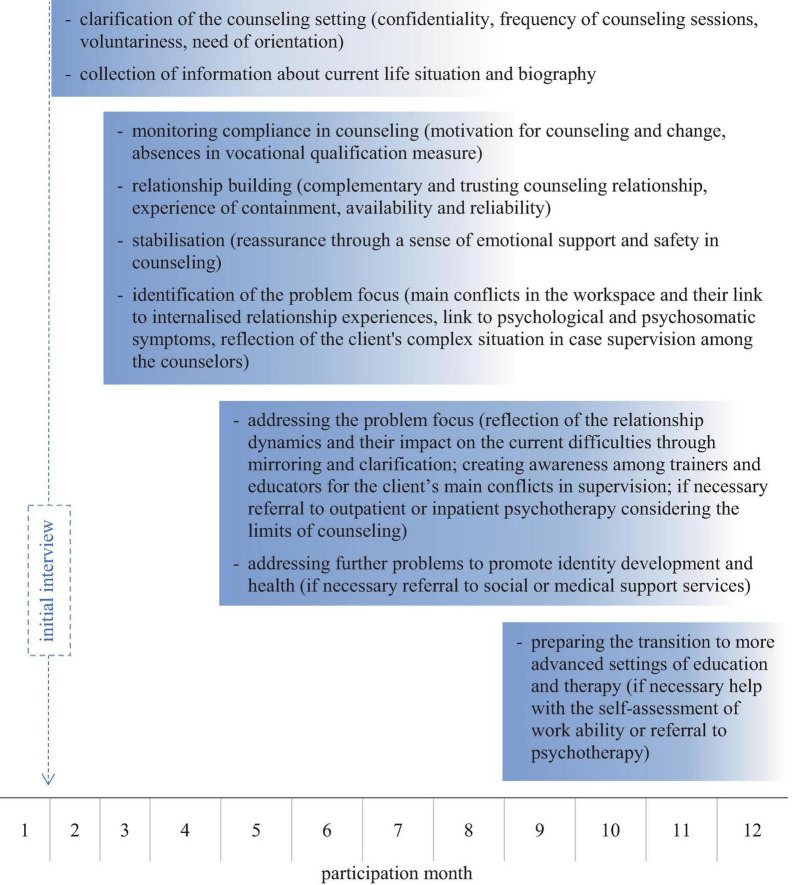
Process of counseling. The figure shows the schematic process of counseling during participation in the vocational qualification measure over one year. Starting with the initial interview, various phases of the process are described. Ideally, all elements of the counseling process are pursued, but this is not always possible.

In the spirit of focal therapy (cf. Balint et al., 2013), the counseling center uses a psychoanalytic approach to help a highly stressed clientele cope emotionally and cognitively with acute personal crises and inner conflicts. In order to promote vocational and personal development, the conflicts brought by the clients are placed at the center of attention (Schnoor, 2011). The extent to which the individual’s biography and the associated problems of identity development lead to any difficulties in professional qualification is also continuously explored together. In this way, the holistic development of the client’s identity becomes the focus of psychoanalytic and social therapeutic counseling practice. Obstacles to vocational qualification are understood as expressions of a conflict dynamic. This dynamic often remains unconscious, but is latently effective and places special demands on educational work in vocational training institutions (Förster-Chanda et al., 2013; Förster-Chanda, 2020; Möhring et al., 2021). More detailled information on the focus and process of the counseling can be found in Figure 1. Internal conflicts are addressed using the basic techniques of psychoanalytic therapy, such as understanding transference and countertransference. This makes it possible to uncover conflictual relationship experiences and create complementary ones.

In the course of crisis management during counseling, the counselors do not limit themselves to the short-term expansion of individual competencies in order to promote professional development and ensure the success of the qualification measure. They also aim to increase the client’s capacity for self-reflection in the long term. The counseling process emphasizes that the causes of one’s own difficulties are not to be found in one’s own personal dynamics, but that general social factors play an equally important role. The social embedding of clients in family, peer and educational structures is of particular importance in the search for causes of individual conflict situations. Thus, psychoanalytically oriented counseling as a low-frequency crisis intervention is linked to social therapeutic forms of intervention that take into account the social embedding of personal relation problems in macrosocietal conditions (Richter, 1978). Psychosocial counseling can help shape the framework of educational institutions if the connection between individual conflicts and macrosocietal conditions is taken into account.

One challenge of the setting is keeping regular appointments with the counselor. This affects the relationship between client and counselor in terms of commitment and a stable framework for counseling sessions. In addition, it is necessary that counselors are clear about what personal issues can be addressed in counseling. Counselors need to point out the limitations of counseling compared to psychotherapy and define the differences with educational goals and support.

It now becomes clear what high demands are placed on the counselors. In order to better meet them, the counseling relationships are continuously reviewed within the framework of psychoanalytically oriented case supervision. Counseling is also flanked by on-site supervision at the training institute with a psychoanalyst and all staff members who have contact with the client to promote multi-professional networking. In addition, counselors complete in-service therapeutic training at therapeutic institutes. On-site, long-term strategies for the care of adolescents are developed together with the specialized teachers and pedagogical staff. For a better understanding of the counseling process, the article shows two case vignettes that underline the individuality of the biographies of participants in vocational training measures.

### 2.2 Process

The counseling process is divided into different phases (Figure 1). First, participants in the vocational qualification measure are invited to an initial interview. There, all of them get to know the counseling offer. During this interview, an initial assessment of their psychosocial situation is made. Following the interview, they are asked to participate in an empirical study to assess their health-related quality of life (the results are presented in Schäfer et al., 2020). Following this, they receive an offer for further counseling sessions. If desired, further counseling appointments are arranged, which vary in number and intensity depending on the circumstances of the individual case. These counseling sessions focus on supporting individual clarification processes, clarifying needs, and readiness for further counseling or therapy in a clinical context. If necessary, the clients are further referred to individually tailored therapeutic services. If indicated, there is also the possibility of a quick consultation in the department for child and family psychosomatics as a cooperating center. If the clearing reveals the motivation to seek further therapeutic help, clients may stay in counseling until the start of outpatient or inpatient therapy.

### 2.3 Clients

Due to the conceptual approach of this article, the participants are described with regard to their need of psychosocial support. A basic systematic assessment of their life situation and mental health using quantitative data can be found in Schäfer et al. (2020, 2022). However, there is no sufficient quantitative data to enable a detailed and differentiated description of these aspects. Therefore, information is used that comes from the long term experience of the counselors in the vocational training institute.

Subjects of this study are young people in the phase of emerging adulthood, who are in a vocational qualification measure at the “Jugendwerkstatt Giessen” (vocational training institute). The day-to-day vocational orientation in the workshops and the qualification of young people in the life phase of emerging adulthood in the training rooms is aimed at those who find themselves without a training contract at the beginning of a new apprenticeship year or without a qualification at the end of compulsory schooling. The working environment in the majority of workshops is characterized by a high proportion of men. Participants are typically hard to reach for institutions like schools and workplaces. Their current life situations and biographies are most often filled with tensions. Frequently occurring psychosocial conflicts and impaired physical and mental health lead together to an impairment of the ability to work. Due to negative experiences in the past, the affected persons are often suspicious of therapeutic offers. They frequently have psychiatric diagnoses from childhood and adolescence that make them feel stigmatized. As a rule, individual problems extend far beyond the problem of lack of qualification. Participants of the vocational qualification measure attribute their current problems to a particular disadvantage they have suffered since childhood in their social environment, but above all in their family of origin. These problems are often based on negative attachment experiences, which make it difficult for young people to enter into lasting social relationships in the private and vocational spheres (such as training contracts lasting several years). The transition from school to work is often a draining challenge, especially for young people with such a biography. As a rule, their educational careers to date have been significantly skewed. A multiple burden of sometimes existential problems is even a condition for participation in the measure. The counselors encounter a wide variety of problems with their clients: neglect, poverty, addiction, school failure, unsecured and/or obstructive housing, early parenthood, psychological and physical suffering, debt and crime.

From 2013 to 2021 a total of 535 participants underwent the vocational qualification measure. Demographic information is shown in Table 1. With regard to participation in initial interviews and counseling, details can only be provided for individual years. This is due to the fact that vocational qualification measures took place over the years and could be started at any time during the year. For example, the initial interview of participants who started the vocational qualification measure at the end of the year might not have taken place until the following year. Furthermore, some participants dropped out of and returned to the vocational training measure, so that initial interviews and counseling could only be offered after a delay.

**TABLE 1:** Demographics of participants.

	Age [years]			Gender [*n* (%)]	
*N*	Range	*M*	SD	Male	Female
535	15–31	20.0	2.7	385 (72%)	150 (28%)

The total number of participants in the vocational qualification measure from 2013 to 2021, their age and gender are displayed.

**FIGURE 2:**
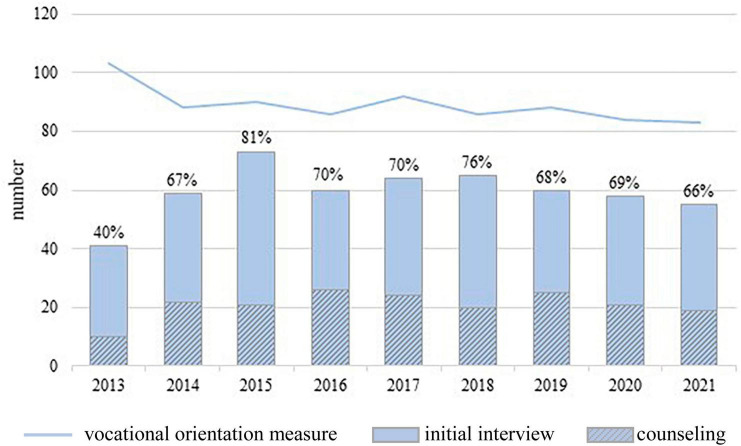
Participation 2013 to 2021. The total number of registered participants in the vocational qualification measure for each year is shown as a line. The percentage of these participants who appeared for an initial interview is represented by the bars and numbers. The striped areas represent the percentage of participants who took advantage of psychosocial counseling following the initial interview. Participants who took part in the measure in more than one year are counted repeatedly.

Figure 2 shows the percentage of participants in the vocational qualification measure, who appeared for an initial interview, respectively, counseling. Over the years, between 39 and 97% (*M* = 69.2%; SD = 14.6%) of the participants in the vocational qualification measure took part in the initial interview. It appears that not all of the participants could be reached for an initial interview. Reasons for this were, for example, a start of the vocational qualification measure at the end of a year, early dropout and limited compliance.

**FIGURE 3:**
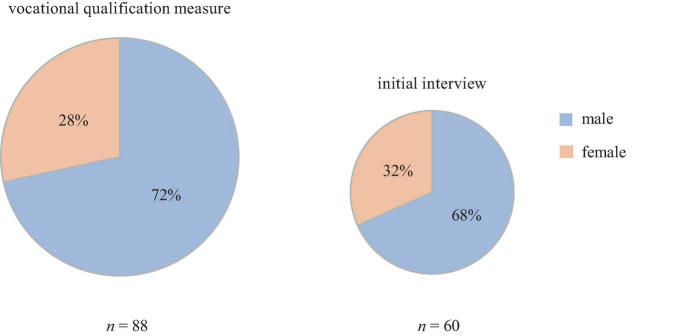
Gender distribution of participants in 2019. The figure illustrates the gender distribution of participants in 2019. In a total of 88 participants in the vocational qualification measure, 63 were male and 25 female **(left side)**. Of the 60 participants who took part in the initial interview, 41 were men and 19 women **(right side)**.

For a closer look at the participation in the initial interview and counseling, 2019 serves as an example (Table 2). Figure 3 shows the gender distribution of participants in 2019. Due to a lack of data, the gender distributions can only be described and not statistically compared. However, it can be noted that the higher proportion of men in the vocational qualification measure remains even when participating in the initial interview. With regard to the existing gender gap in the utilization of psychosocial support services (Rommel et al., 2017), this result shows the importance of the institutional context for the utilization of psychosocial assistance.

**TABLE 2:** Participation in 2019.

	Vocational qualification measure	Initial interview	Counseling	Referral
Number of participants	*N* = 88	*n* = 60	*n* = 25	*n* = 6
Percentage of all participants (%)		68	28	7

The table shows the number of participants in the vocational qualification measure in 2019 who could be reached by the counseling offer (initial interview, counseling, referral).

## 3 Detail to understand programmatic key elements

The relationship between counseling and personal development in the school-to-work transition is illustrated by two case-vignettes.

### 3.1 Case vignette–Tom

Tom (18) shows a self-confident appearance and has a muscular body. He is humorous and friendly. From the very first encounter it is clear that he is highly sensitive to what he perceives to be unfair. The initial interview is a mere exchange of information. Tom had been assigned to the metal workshop. There he has made good contact with other participants and was enthusiastic about the metal work. A social pedagogue reported to the counselor that conflicts had arisen between him and his instructors. Tom had become very angry when he was prevented from charging his cell phone while an instructor did so as a matter of course. Then he also had difficulty with instructions that he felt were contradictory depending on which instructor gave them to him. The social pedagogue who initiated our first appointment suspected that he was transferring his relationship with his father to the instructor.

Tom is the third of seven children. His parents have lived apart since childhood and he was growing up in juvenile facilities and alternating with his father, who is an alcoholic and often violent. His younger siblings live with his father. There, he takes over the father’s child-rearing responsibilities such as homework supervision, meal preparation, and grocery shopping. Despite several changes of residence, he manages to graduate from secondary school and subsequently he takes part in the vocational qualification program. He likes to come to the workshop frequently and also would like to stay longer to escape the stressful situation and the threat from his father at home. He does not want to turn to youth welfare because he fears that his siblings would have a similarly hard time in the youth welfare facilities. In the course of counseling, it turns out that Tom generally has a hard time tolerating inconsistent or even unfair behavior from adults. After about 3 months in the vocational qualification program, he becomes an apprentice. There he works side by side with his ex-partner. The mixture of mutual private resentment and the desire for support in achieving the training goals overwhelms Tom. With the help of counseling, Tom manages to set boundaries for his father in an argument. From this point on, his father stops physically abusing him. He has moved into a shared apartment and starts training to become a nursing assistant. Understanding Tom’s inner conflict, as evidenced by his conflicts with instructors, and reflecting on difficult decisions in counseling enables Tom’s transition from school to work.

### 3.2 Case vignette–Carla

Carla (20) wears work clothes, has several piercings and makes an overall well-groomed impression. She is friendly and somewhat reserved. She has a likeable charisma. She initially attends the vocational qualification measure on the basis of the integration agreement with the job center without any real goal. After some time, she takes a liking to the work in the wood workshop and appears more regularly. She also accepts the offer of psychosocial counseling because of the good contact with the counselor. At the beginning, the focus is on her cannabis use. She has high absenteeism and cannot take advantage of the services offered at the vocational training. Her two daughters have been in the care of various foster families for almost three years. She is a frequent visitor there and hopes to one day be able to live with her children again. Due to her drug use, she was no longer able to adequately care for the children and the Youth Welfare Office became aware of her. Seeing the children again is her constant motivation to change. However, she has been addicted to drugs for more than ten years and managed to withdraw from harder drugs by attending an addiction clinic shortly before she started working at the vocational training institute. As an adolescent, she was placed in several inpatient psychiatric facilities and survived serious suicidal attempts. She is severely traumatized due to early childhood at the hands of her abusive father. Today, she suffers from being the only one of four children not to have a career and being ridiculed by her siblings for her mental illness, when they would lovingly care for her depressed mother.

She uses the counseling situation primarily as a venue for trusting conversations that eventually give her enough confidence to stop her cannabis use. Feelings that had not found a place inside her for years come to the surface. With the help of the counseling sessions, she learns to deal with them and gradually gains more confidence in herself. After almost a year in the vocational qualification program, she finally signs a three-year training contract in a specialist trade. She sees her children regularly at meetings accompanied by the youth welfare office.

### 3.3 Reflection of the case vignettes

Vaillant’s (2012) theory on the connection between the prevalence of immature defenses and vocational failure or low social class in adulthood can be illustrated in the presented case vignettes Tom and Carla. Tom uses immature defense mechanisms such as splitting into good and bad, projecting unconscious experiences into current relationship partners, and denying incompatible current perceptions. He seems to maintain a splitted image of his parents as good mother and bad father, to project his threatening and neglecting father image onto instructors and supervisors, and to deny his disfunctional living situation by reporting it in a humorous way. These mechanisms result in a neurotically entangled life situation with the father and siblings, to major problems at school and later to a complicated and difficult path to pursue a professional career. Due to her negative relationship experiences in form of abuse, rejection and devaluation, Carla shows immature defense mechanisms in form of acting out, projection and devaluation of self-image. For example, she uses drugs to suppress overwhelming negative and offending feelings, lacks trust in others and herself, and appears to have introjected the negative image her family ascribes to her. Due to her immature defenses, she falls into a psychologically unstable state, has suicide attempts, loses custody of her children, and has no high school diploma or career path before entering the vocational training institute where she works out her personal path to apprenticeship.

The path into the phase of growing up according to Erikson and those who continue to research in his sense certainly describes much of the situation of the two participants. Their attempt to find a stable orientation in their professional lives does not stand on its own, but is connected with the readjustment of their relationship to their families and places of growing up, with the search for a lasting partnership and the completion of puberty, which lead to an increased self-confidence. Nevertheless, unresolved personal trauma in earlier stages of identity development continue to influence the current situation of both clients and mark limits to their personal development. However, this limitation of development is two-sided. The scope for young adults’ personal development depends both on the opportunities afforded them by adult workers and on the institutional environment to which they belong (King, 2022).

## 4 Discussion: reached by the counseling

The title of this section points at the proximity of the described concept of psychosocial counseling for participants in a vocational qualification measure in the life phase of emerging adulthood. It is important for participants to achieve the skills they need to begin with an apprenticeship. If this aim remains out of reach it is required to counteract the ongoing chronification of their personal problems and inner conflicts. The encounter presented here blurs the lines between psychosocial counseling and political programs of social integration into the labor market. This specificity of the counseling setting is discussed below.

The counseling offer has reached about a quarter of the participants in vocational qualification measures over the years. An improvement in opportunities in the school-to-work transition can be demonstrated in many counseling processes. The opportunity for self-reflection, the increase of self-confidence and stability through regular contact with a trusted person, are to be mentioned as direct personal gains through participation in the service. The professional background of the counselors usually counteracts the further consolidation of chronic psychological stress. This is because psychosocial counseling represents a therapeutic approach to mental health problems that have already persisted for a long time in a low-threshold, non-clinical setting. Contact with therapeutic settings is sometimes difficult for clients due to bad experiences in the past. Two thirds of the participants were reached by the initial interview. The low-threshold offer of psychosocial counseling might be especially important for men, as clinical services in Germany are more often used by women (Rommel et al., 2017). In 2019 65% of the male participants in the vocational qualification measure took part in an initial interview compared to 75% of the female participants. A strength of the project is the opportunity to reach these men from the unprivileged working class milieu. In counseling, destabilization through confrontation with inner conflicts is encountered by the possibility of using a longer period of time (12 months) compared to common counseling services. This time period also allows a deeper understanding of the impact of the mental health problems on transition from school to work in each individual case. On-site multiprofessional cooperation increases the awareness of the issue of mental stress among social pedagogues and instructors. Nevertheless, there are difficulties and limits of the counseling service:

• Treatment of mental health problems: Without referral to a clinical setting, clients do not receive clinical diagnosis. While this prevents stigmatizing, it also leaves out opportunities of services that are based on clinical diagnosis. Less than a tenth of the participants could be placed in outpatient or inpatient therapy during counseling. In this case, not only the higher threshold of psychotherapy plays an important role, but also the good relationship with the counselor that develops in the course of counseling. They might understand psychotherapy as a continuation of counseling, which, however, offers more possibilities in dealing with emotional conflicts.
• Compromising: Problems at the workshop are addressed in the counseling. Working through these conflicts becomes more difficult because of the proximity and cooperation between the counseling center and the vocational training institute. On the one hand, this has a positive effect on the cooperation between the social pedagogues and the counselors. On the other hand, this may also lead to role diffusion. A lack of protection of the counseling setting occurrs repeatedly because of the proximity. Counselors may be personally involved with instructors and social pedagogues through the shared work context. Also, several clients in one workshop often have the same counselor. It is possible that these circumstances limit clients’ perceived freedom to talk about problems with instructors or participants during counseling, despite the promise of confidentiality. However, due to comparable settings, these difficulties also occur in clinical day-care or inpatient settings.
• Conflicting goals: The counseling sometimes leads to a shift in focus from educational work to psychosocial problems among the clients themselves, but also in the institution.

## 5 Conclusion

In the psychosocial counseling at a vocational training institute, individuals with severe mental health problems who are resistant to psychotherapy in a clinical setting, are given an impression of the benefits of psychotherapeutic intervention. The acceptance of the counseling offer as well as the obstacles in referral to psychotherapeutic services show that participants in vocational qualification measures need a low-threshold intervention offer. In a multiprofessional network, insights into the complex nature and dynamics of psychological processes are gained on site, leading to an improvement in the life situation of socially disadvantaged emerging adults. Far more research is needed to understand what offers can reach this lower social class group for psychotherapeutic help.

## Data availability statement

The original contributions presented in this study are included in this article/supplementary material, further inquiries can be directed to the corresponding author.

## Ethics statement

The studies involving humans were approved by the Ethics Committee at the Department of Medicine at the Justus Liebig University of Giessen. The studies were conducted in accordance with the local legislation and institutional requirements. Written informed consent for participation in this study was provided by the participants’ legal guardians/next of kin. Written informed consent was obtained from the individual(s) for the publication of any potentially identifiable images or data included in this article.

## Author contributions

All authors listed have made a substantial, direct, and intellectual contribution to the work and approved it for publication.
